# Supplementary material for: CoCas9 is a compact nuclease from the human microbiome for efficient and precise genome editing
Source: Nat Commun. 2024 Apr 24;15:3478. doi: 10.1038/s41467-024-47800-9 (PMC11043407; doi:10.1038/s41467-024-47800-9)
Supplement: Supplementary file 1 — Supplementary Information [file 41467_2024_47800_MOESM1_ESM.pdf]

SUPPLEMENTARY INFORMATION

CoCas9 is a compact nuclease from the human microbiome for efficient and precise genome editing

Eleonora Pedrazzoli[1#], Michele Demozzi[1#], Elisabetta Visentin[1#], Matteo Ciciani[1], Ilaria Bonuzzi[1], Laura Pezzè[2], Lorenzo Lucchetta[1], Giulia Maule[1], Simone Amistadi[1,3], Federica Esposito[4], Mariangela Lupo[4], Annarita Miccio[3], Alberto Auricchio[4,5], Antonio Casini[2], Nicola Segata[1*], Anna Cereseto[1*]

Affiliations:

[1] Department of Computational, Cellular and Integrative Biology (CIBIO), University of Trento, 38123 Trento, Italy

[2] Alia Therapeutics, Trento, Italy

[3] Université de Paris, Imagine Institute, Laboratory of chromatin and gene regulation during development, INSERM UMR 1163, Paris, France

[4] Telethon Institute of Genetics and Medicine (TIGEM), 80078 Pozzuoli (NA), Italy

[5] Medical Genetics, Department of Advanced Biomedical Sciences, University of Naples "Federico II", 80131 Naples, Italy

# These authors contributed equally
* These authors jointly supervised this work

Correspondence should be addressed to: anna.cereseto@unitn.it and nicola.segata@unitn.it

Supplementary Figure 1

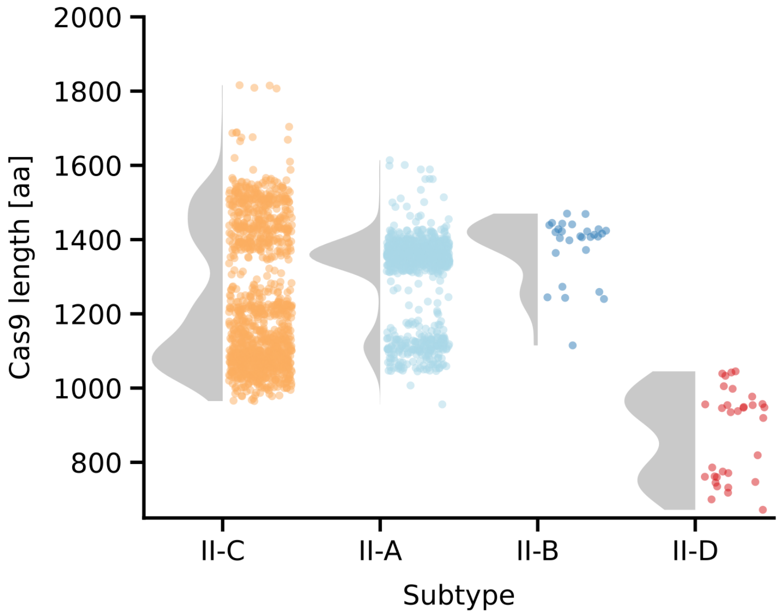

Supplementary Figure 1. Distribution of Cas9 length.

Size distribution of all the identified Cas9 proteins in **Figure 1**, divided by subtype. The length of subtype II-A and II-C Cas9 proteins follows a bimodal distribution with two peaks around 1100 aa and 1400-1500 aa, as previously reported[1]. II-C: n=1185; II-A: n=961; II-B: n=28; II-D: n=32. Source data are provided as a Source Data file.

Supplementary Figure 2

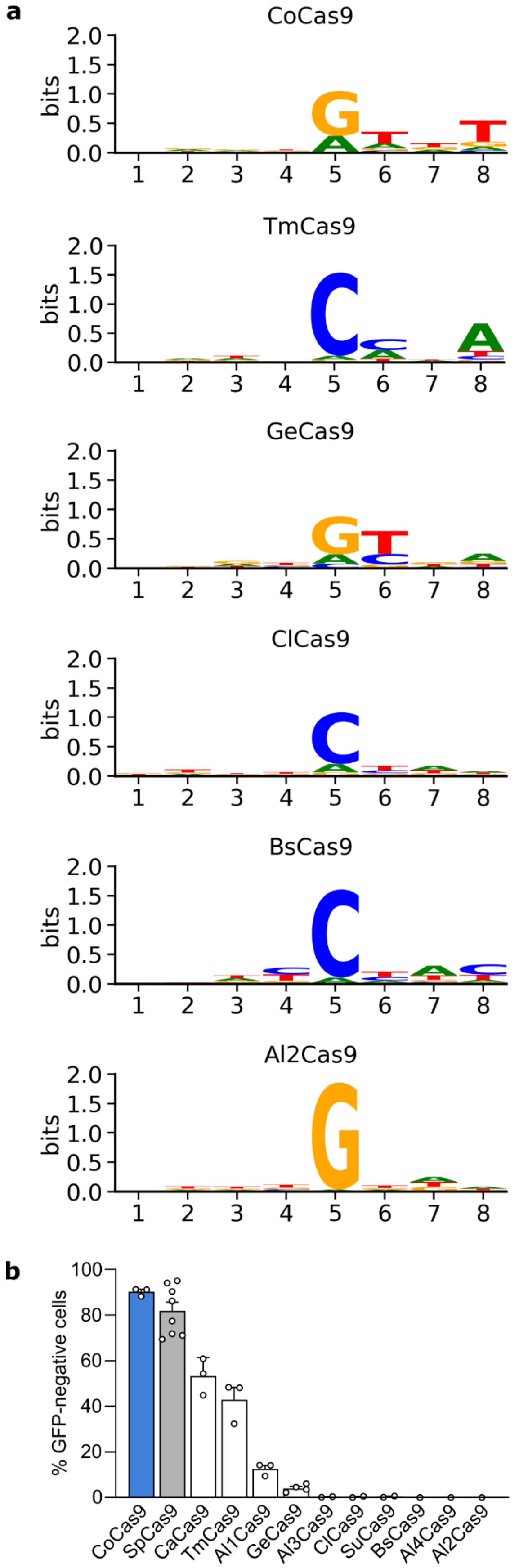

Supplementary Figure 2. PAM sequences of selected Cas9 orthologs identified in the microbiome database by Pasolli et al.[2]

(**a**) The PAM sequences identification and derived logos were obtained as described in Methods. (**b**) Activity of 10 newly identified Cas9 orthologs and the benchmark nuclease SpCas9 compared through an EGFP disruption assay in U2OS cells; data reported as mean ± SEM for $n \geq 3$ independent experiments. Source data are provided as a Source Data file.

Supplementary Figure 3

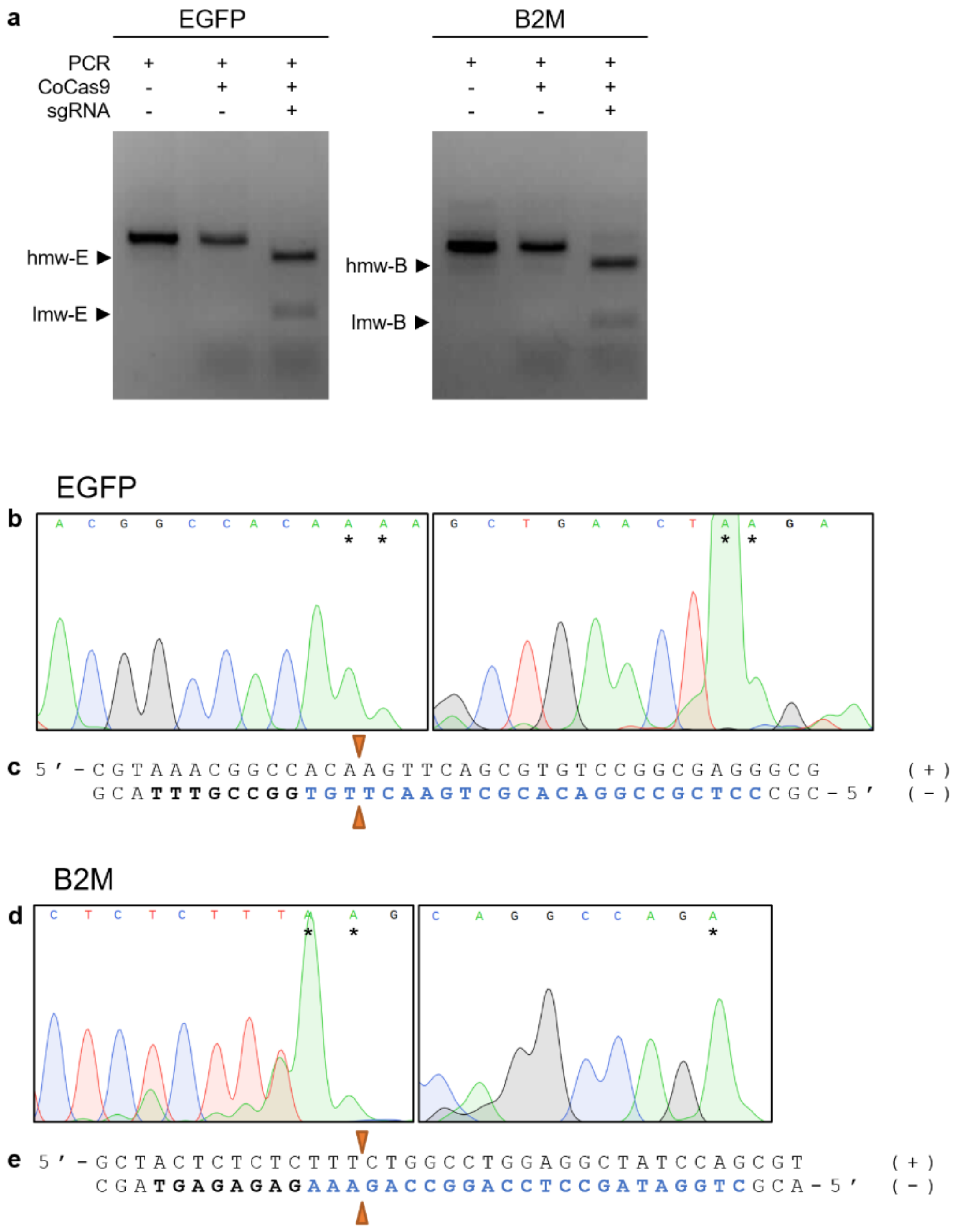

Supplementary Figure 3. CoCas9 cleavage pattern identification.

(**a**) Agarose gel showing EGFP and B2M PCR fragments obtained from the *in vitro* cleavage. (**b**) The EGFP cleavage products (high and low molecular weight bands, hmw-E and lmw-E from panel a) were Sanger sequenced. Left panel: chromatogram of the hmw-E (plus strand), right panel: chromatogram of the lmw-E (negative strand). (**c**) Scheme of CoCas9 cleavage in the EGFP target. (**d**) Left panel: chromatogram of the B2M cleavage product (hmw-B from panel a)t; right panel: chromatogram of the B2M lmw-B from panel a). (**e**) Scheme of CoCas9 cleavage in the B2M target. In (**b** and **d**) asterisks indicate adenines (A) produced as byproducts by Sanger sequencing. In (**c** and **e**) the orange triangles represent the cut site, the protospacer sequence is in blue and the PAM is in bold. Sequencing primers in **Supplementary Data 6**. Source data are provided as a Source Data file.

Supplementary Figure 4

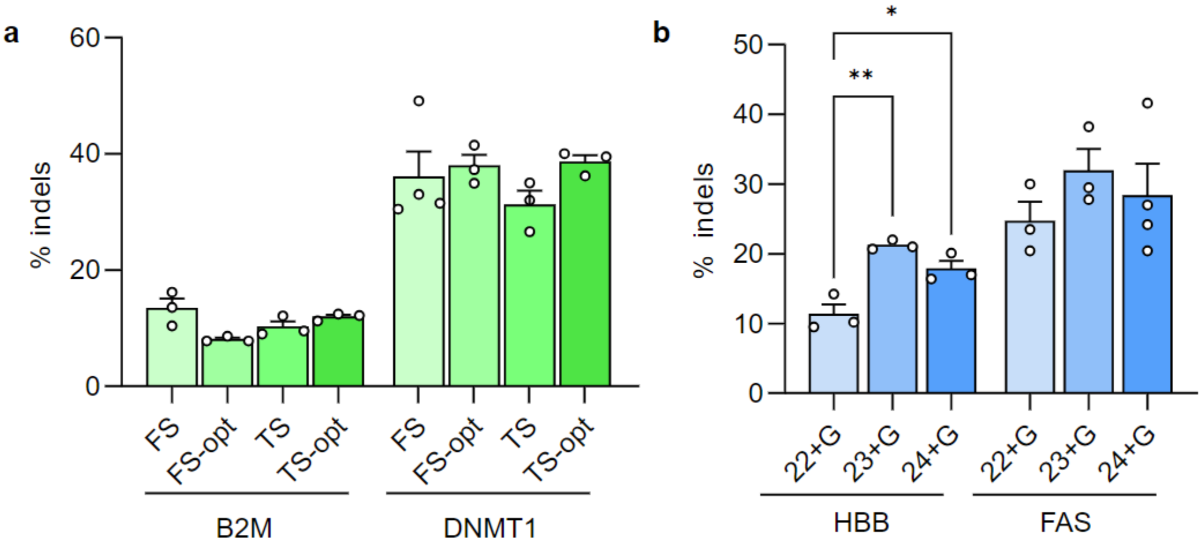

Supplementary Figure 4. Spacer length preference and CoCas9 sgRNA scaffold optimization (**a**) Side-by-side comparison of alternative CoCas9 sgRNA scaffolds. CoCas9 full scaffold (FS), obtained by direct repeat and anti-repeat fusion through a GAAA tetraloop, was compared with three alternative sgRNA designs. The first version contains base substitutions aimed at increasing the stability of its secondary structure (FS-opt), for the second one we shortened the repeat:anti-repeat loop as previously reported[3,4] (TS), and the third corresponds to a stabilized version of the trimmed scaffold (TS-opt). The editing activity was tested in two endogenous genomic loci (*B2M* and *DNMT1*). (**b**) The optimal sgRNA spacer length for CoCas9 was assessed by targeting *HBB* and *FAS* genes in HEK293T cells using spacers ranging from 22 to 24 bp. Each spacer contained an appended extra 5’ G for efficient transcription from the U6 promoter. Data plotted as mean ± SEM for n≥3 biological replicates. Statistical significance was assessed with a one-way ANOVA followed by a two-sided Holm-Šídák test; **P<0.01, *P<0.05, not significant comparisons not shown. Source data are provided as a Source Data file.

Supplementary Figure 5

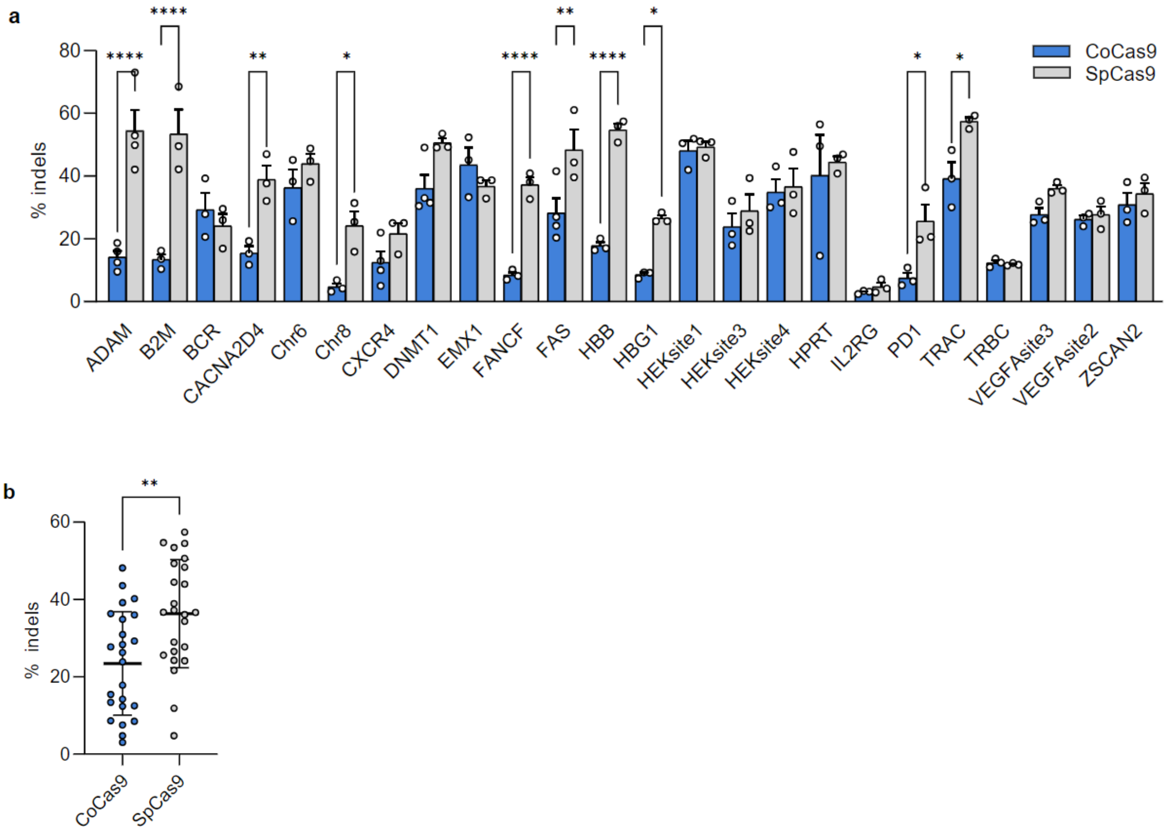

Supplementary Figure 5. Evaluation of CoCas9 activity in mammalian cells.

(a) Side-by-side comparison of the editing activity of CoCas9 and SpCas9 on a panel of 24 genomic loci in HEK293T cells using overlapping spacers. Data plotted as mean ± SEM for n≥3 biological replicates. (**b**) Dot plot summary of the editing efficiency of CoCas9 and SpCas9 at the n=24 loci from panel (a). Editing efficiency was evaluated via TIDE analysis. In panel (**a**), the statistical significance was assessed using a two-way ANOVA corrected for multiple comparisons using the Holm-Šídák method. In panel (**b**) the statistical significance was assessed using a two-sided t-test. ****P<0.0001, ***P<0.001, **P<0.01, *P<0.05, not significant comparisons not shown. Source data are provided as a Source Data file.

Supplementary Figure 6

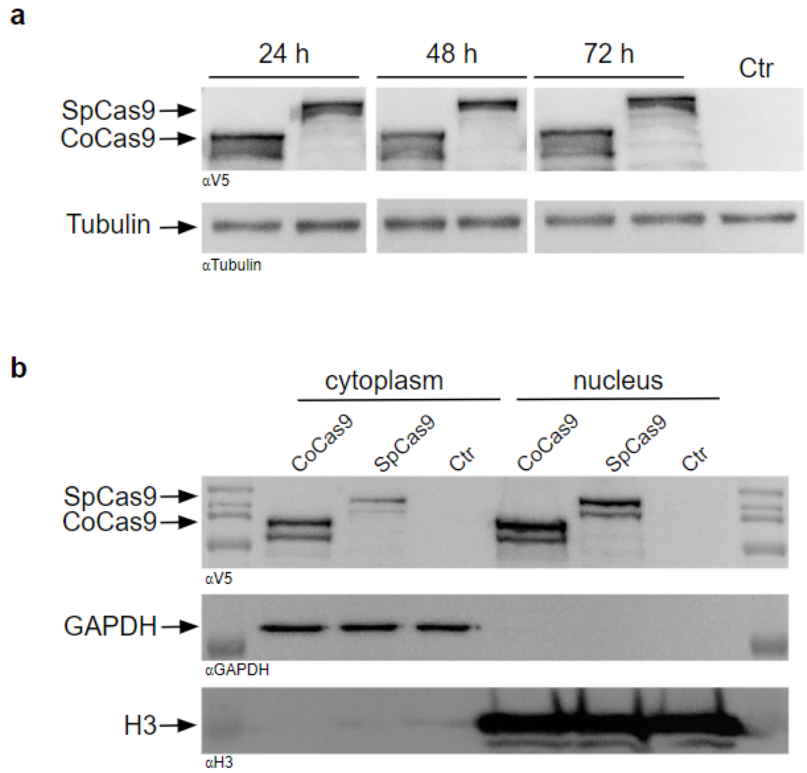

Supplementary Figure 6. Comparison of CoCas9 and SpCas9 expression levels. (**a**) Western blot analysis from whole cell extracts of HEK293T cells transfected with pX-SpCas9 or pX-CoCas9 at three different timepoints and probed with the anti-V5 antibodies to detect CoCas9 and SpCas9 fused to V5 tags, and anti-Tubulin for loading normalization. (**b**) Western blot analysis from cytoplasmic or nuclear fractions (see **Methods**) of HEK293T cells 72 hours after transfection with pX-SpCas9 or pX-CoCas9 plasmids. Cas proteins were detected with anti-V5 antibodies; anti-GAPDH or anti-H3 were used as controls of cytoplasmic or nuclear fraction respectively. Ctr=non-transfected cells. Source data are provided as a Source Data file.

Supplementary Figure 7

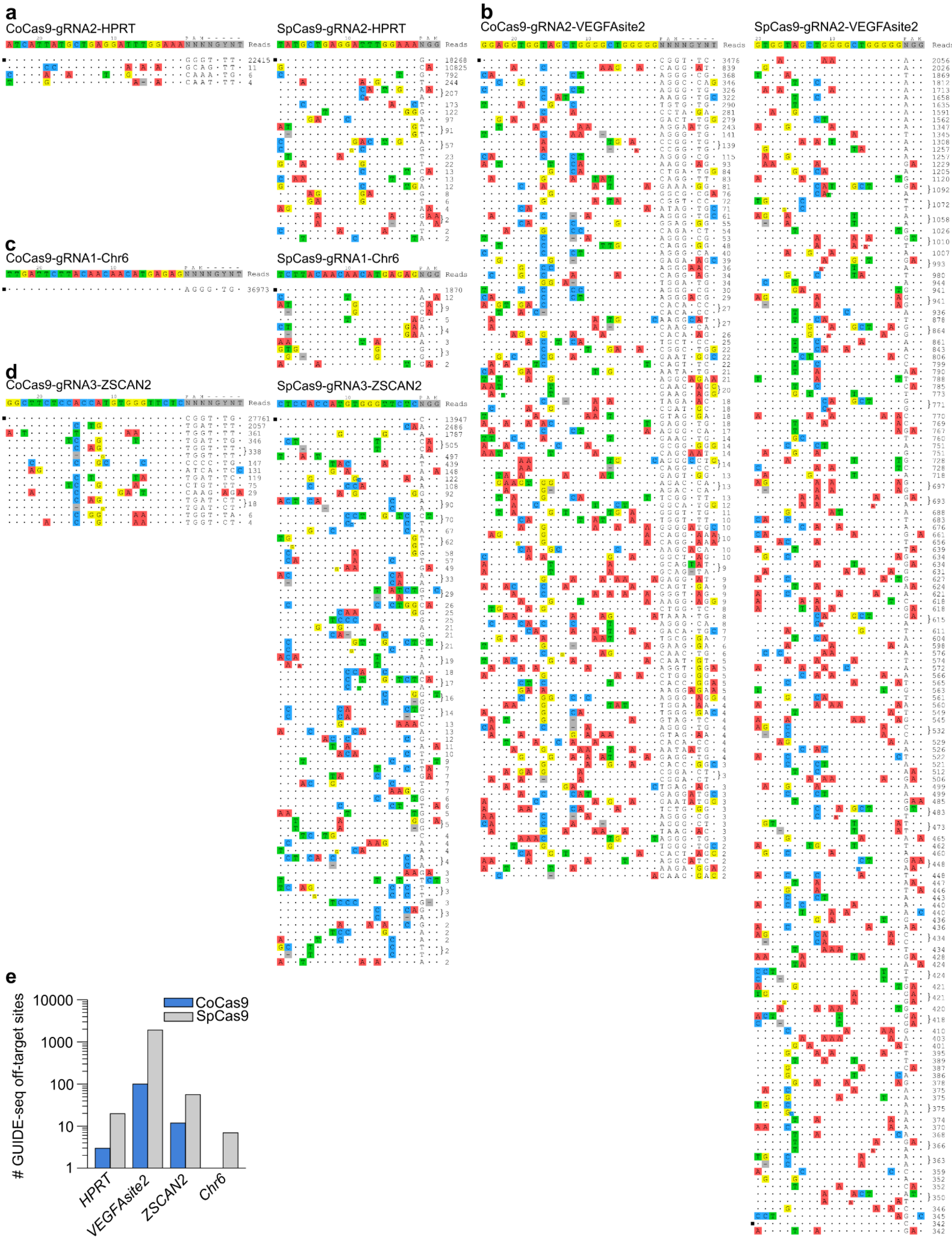

Supplementary Figure 7. CoCas9 and SpCas9 comparative off-target analysis. OT sites detected by GUIDE-seq with CoCas9 and SpCas9 targeting the genomic loci *HPRT* (**a**), *VEGFAsite2* (**b**), *Chr6* (**c**), *ZSCAN2* (**d**). For SpCas9 *VEGFAsite2* in (**b**) 135 off-target sites are reported out of a total 1815 identified sites. Each detected OT is accompanied by its number of GUIDE-seq reads, which is an indirect measure of the propensity of the site to be cleaved by the nucleases. Black squares indicate the ON-target site. (**e**) Total number of OT sites obtained with CoCas9 and SpCas9 from GUIDE-seq in panels (**a-d**). Source data are provided as a Source Data file.

Supplementary Figure 8

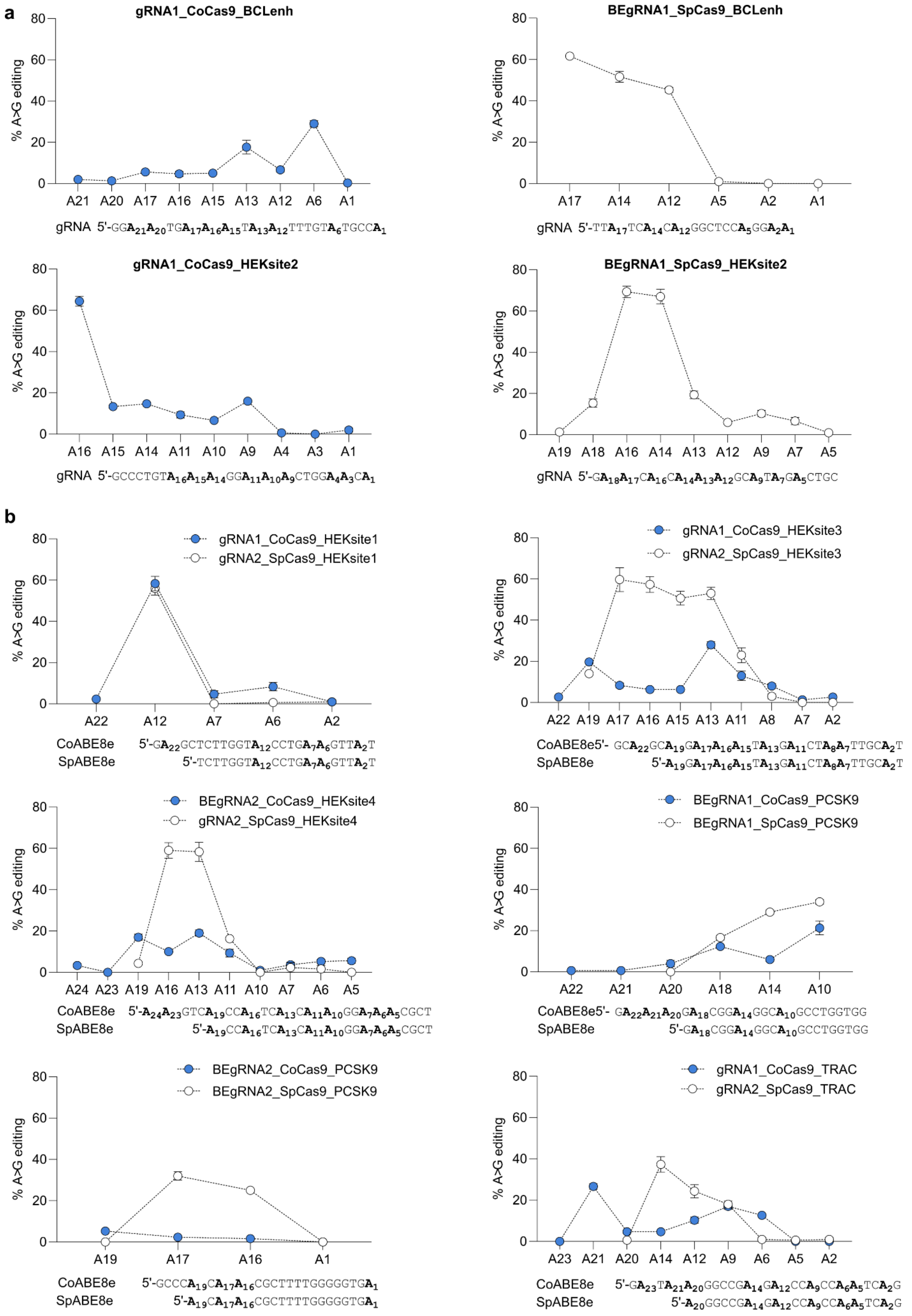

Supplementary Figure 8. Comparison of base editing efficacy between CoCas9 and SpCas9 adenine base editors.

(**a**) Base editing efficiency of CoABE8e and SpABE8e at the indicated loci using non overlapping sgRNAs. The A residues are counted starting from the PAM proximal side of the spacer going in the 5' direction. (**b**) Base editing efficiency of CoABE8e and SpABE8e using overlapping PAM at the indicated loci. The A residues are counted starting from the PAM proximal side of the spacer going in the 5' direction. Data reported as mean ± SEM for n=3 biological replicates. Source data are provided as a Source Data file.

Supplementary Figure 9

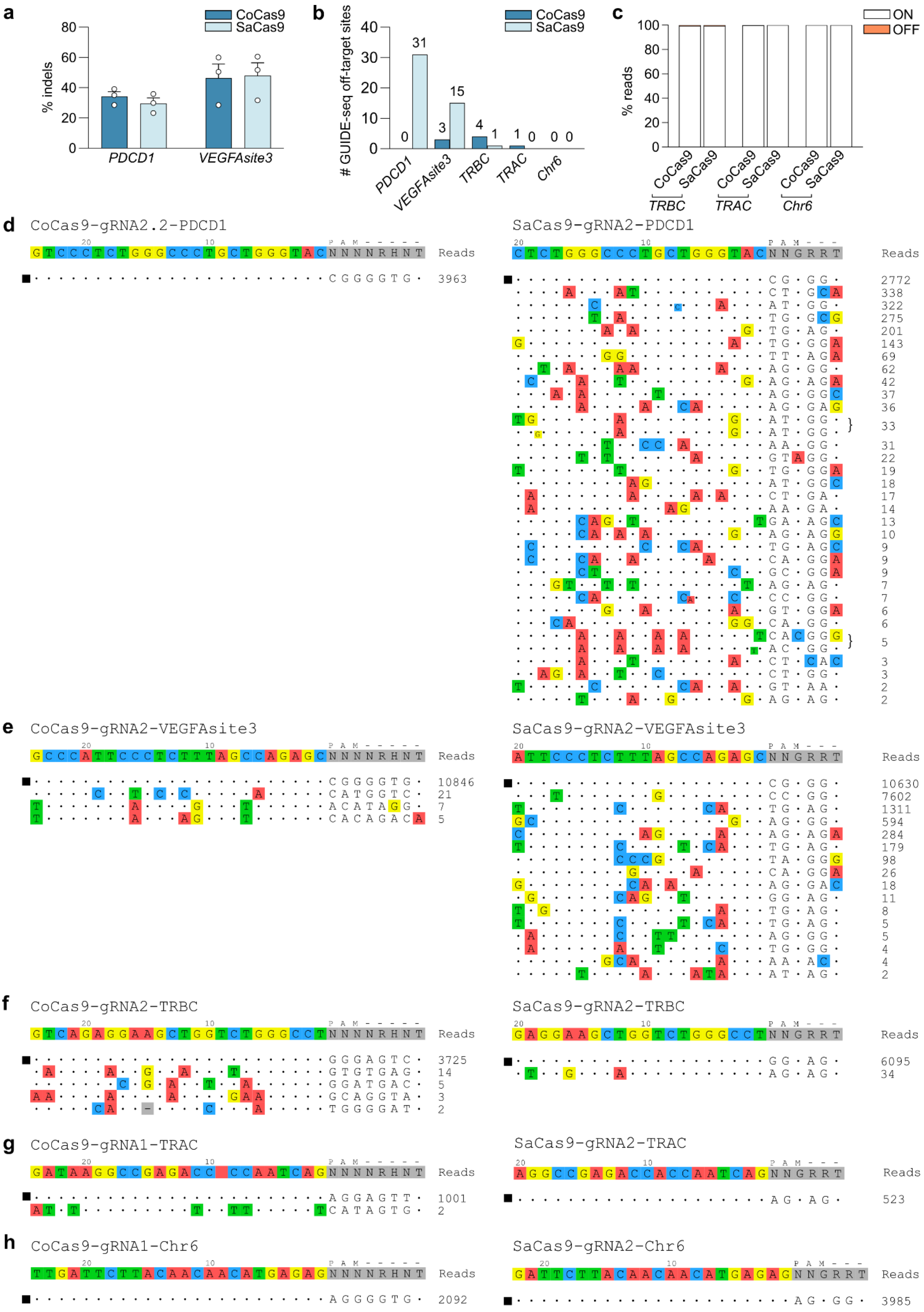

Supplementary Figure 9. CoCas9 and SaCas9 comparative off-target analysis. (**a**) Editing efficiency of CoCas9 and SaCas9 using overlapping sgRNAs targeting *PDCD1* and *VEGFAsite3* (see panels **d** and **e**). Values are represented as points, error bars represent mean ± SEM of n=3 biological replicates for each site. (**b**) Total number of GUIDE-seq-detected OT sites for CoCas9 and SaCas9 for each locus tested in panels (**d-h**). (**c**) Comparison of CoCas9 and SaCas9 percentages of ON and OFF-target GUIDE-seq reads at *TRBC*, *TRAC* and *Chr6* loci. (**d-h**) OT sites detected by GUIDE-seq with CoCas9 and SaCas9 targeting the genomic loci *PDCD1* (**d**), *VEGFAsite3* (**e**), *TRBC* (**f**), *TRAC* (**g**) and *Chr6* (**h**). Each detected OT is accompanied by its number of GUIDE-seq reads, which is an indirect measure of the propensity of the site to be cleaved by the nucleases. Black squares indicate the ON-target site. Source data are provided as a Source Data file.

Supplementary Figure 10

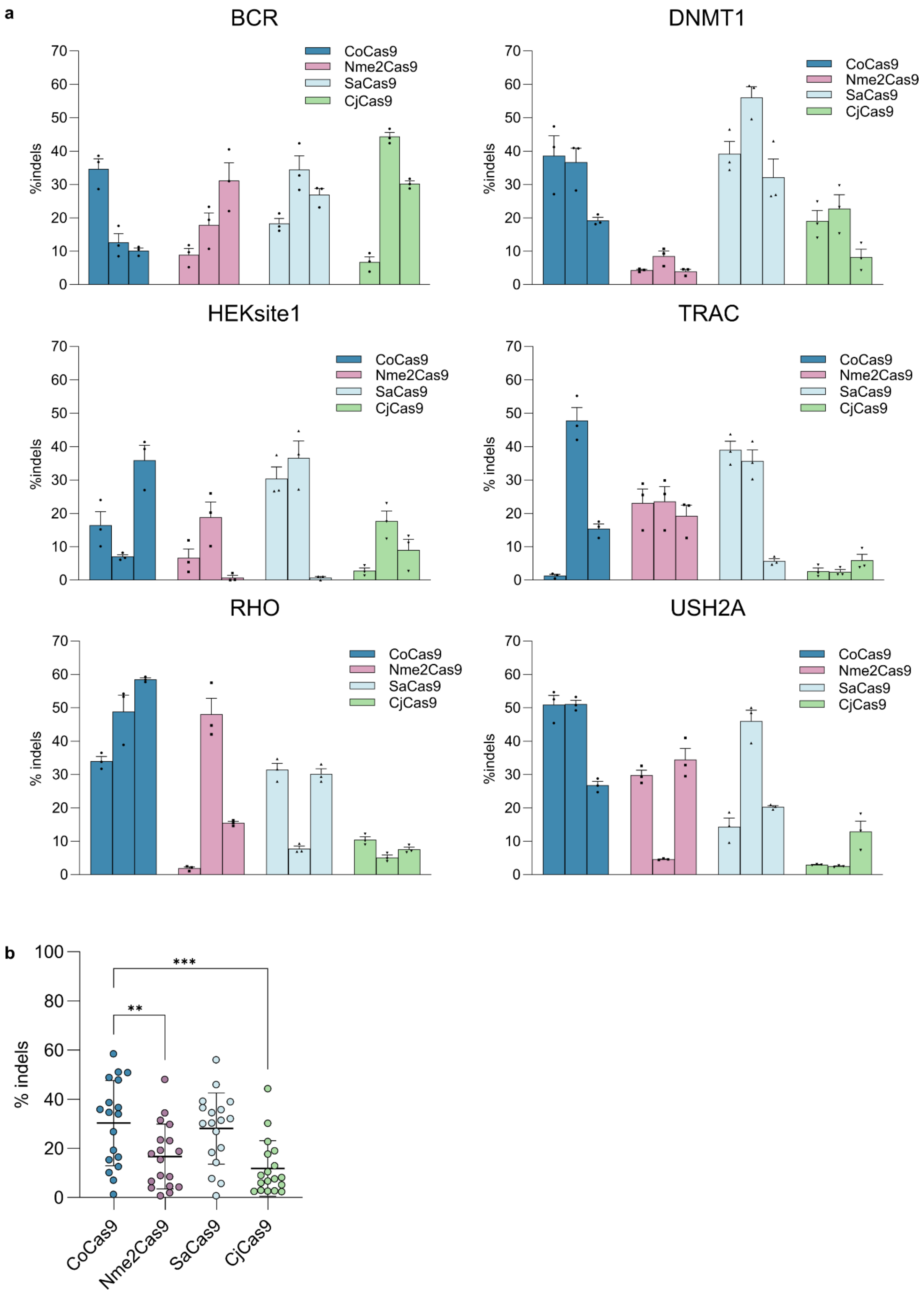

Supplementary Figure 10. Comparison of nuclease activity among CoCas9 and other compact Cas9s.

(**a**) Nuclease efficiency of CoCas9, Nme2Cas9, SaCas9 and CjCas9 at the indicated loci. In all of them, 3 non-overlapping sgRNAs were used for each Cas9 (sequences of each target protospacer and PAM listed in **Supplementary Data 2**). Dots represent values and error bars represent mean ± SEM of n=3 biological replicates. (**b**) Summary dot plot generated from data in (**a**). Statistical significance was assessed using a one-way ANOVA followed by a two-sided Holm-Šídák test; ***P<0.001, **P<0.01, *P<0.05, not significant comparisons not shown. Source data are provided as a Source Data file.

Supplementary Figure 11

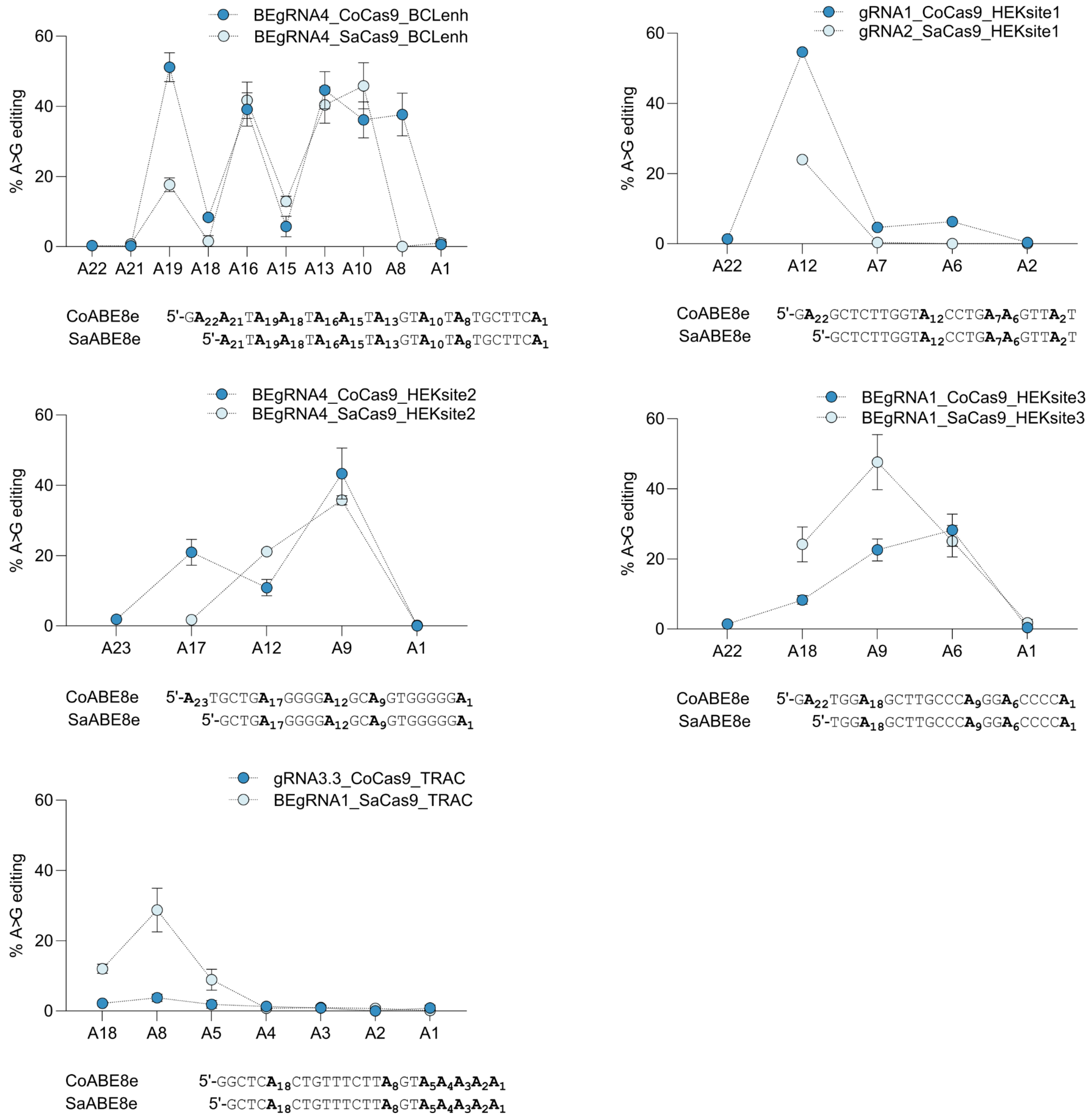

Supplementary Figure 11. Comparative base editing efficacy between CoCas9 and SaCas9 adenine base editors. Base editing efficiency of CoABE8e and SaABE8e at the indicated loci using overlapping sgRNAs (target protospacer and PAM reported in **Supplementary Data 2**). The A residues are counted starting from the PAM proximal side of the spacer going in the 5' direction. Data reported as mean ± SEM for n=3 biological replicates. Source data are provided as a Source Data file.

Supplementary Figure 12

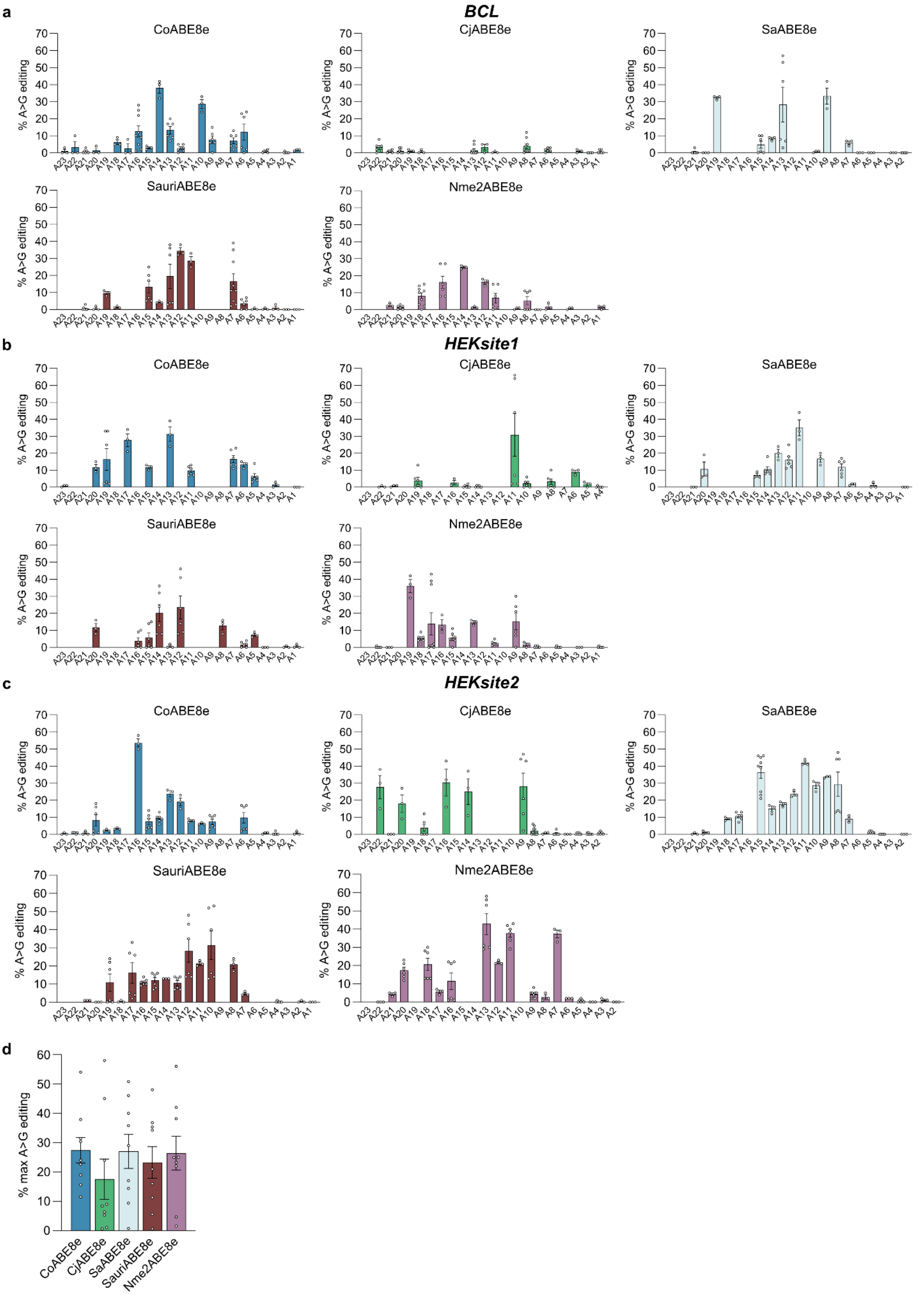

Supplementary Figure 12. Base editing activity window and efficiency of compact ABEs.

(**a-c**) Base editing activity window for CoABE8e, CjABE8e, SaABE8e, SauriBAE8e and Nme2ABE8e at BCL, HEKsite1 and HEKsite2; non-overlapping sgRNAs were used (9 total sgRNAs for each ABE, target protospacer and PAM reported in **Supplementary Data 2**). The A residues are counted starting from the PAM proximal side of the spacer going in the 5' direction. Values are represented as points, error bars represent mean ± SEM of n≥3 biological replicates for each site, with each position representing 1–3 genomic sites. (**d**) Summary of the highest A>G conversion for each sgRNA in (**a-c**). Values are represented as points, error bars represent mean ± SEM of n≥3 biological replicates for each site, with each position representing 1–3 genomic sites. Source data are provided as a Source Data file.

Supplementary Figure 13

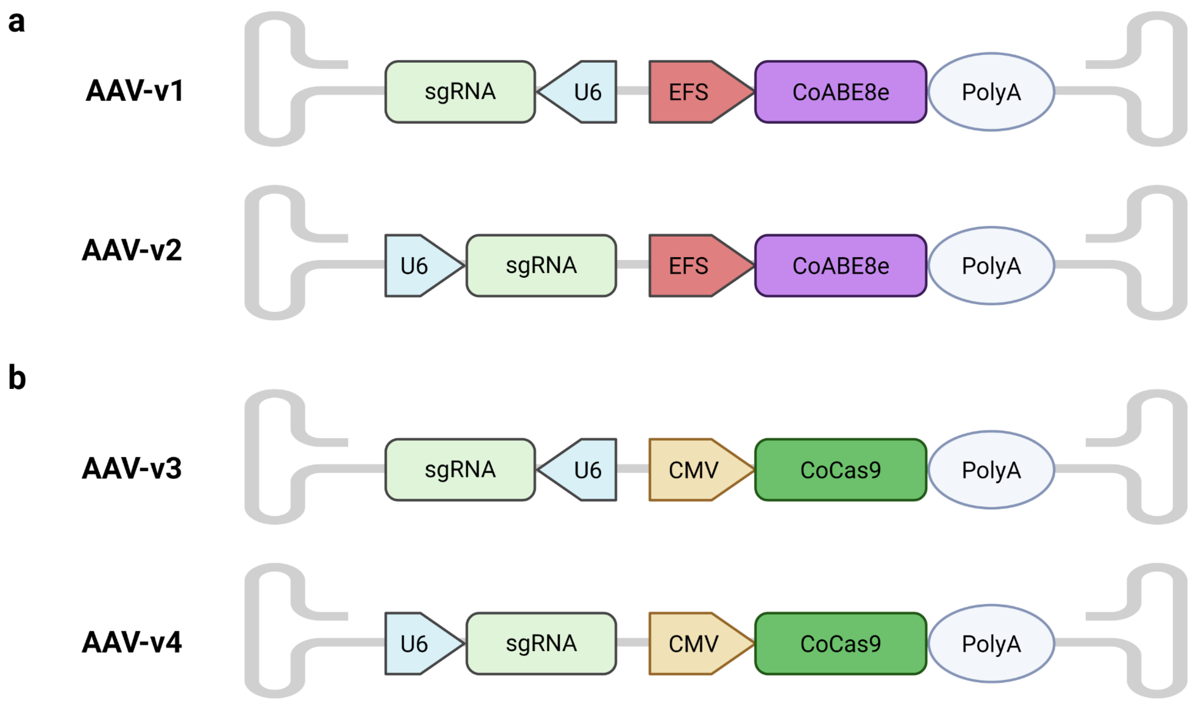

Supplementary Figure 13. Representation of AAV constructs containing CoCas9.

(**a**) Schematic of two designs of the all-in-one AAV vector expressing CoABE8e under the EFS promoter and the gRNA1_CoABE8e_HEKsite2 under the U6 promoter. The results relative to these constructs are reported in **Figure 5d-e**. (**b**) Schematic of two designs of the all-in-one AAV vector expressing CoCas9 under the CMV promoter and gRNA3.1_CoCas9_RHO under the U6 promoter. The AAV-v3 design is also reported in **Figure 5f**. The target protospacers and relative PAM are reported in **Supplementary Data 2.** Created with BioRender.com.

REFERENCES

1. Gasiunas, G. *et al.* A catalogue of biochemically diverse CRISPR-Cas9 orthologs. *Nat. Commun.* **11**, 5512 (2020).
2. Pasolli, E. *et al.* Extensive Unexplored Human Microbiome Diversity Revealed by Over 150,000 Genomes from Metagenomes Spanning Age, Geography, and Lifestyle. *Cell* **176**, 649–662.e20 (2019).
3. Cong, L. et al. Multiplex genome engineering using CRISPR/Cas systems. Science 339, 819–823 (2013).
4. Ran, F. A. et al. In vivo genome editing using Staphylococcus aureus Cas9. Nature 520, 186–191 (2015).
