# Supplementary material for: CoCas9 is a compact nuclease from the human microbiome for efficient and precise genome editing
Source: Nat Commun. 2024 Apr 24;15:3478. doi: 10.1038/s41467-024-47800-9 (PMC11043407; doi:10.1038/s41467-024-47800-9)
Supplement: Supplementary file 3 — Description of Additional Supplementary Files [file 41467_2024_47800_MOESM3_ESM.pdf]

Description of Additional Supplementary Files

File Name: Supplementary Data 1
Description: Sequences of the sgRNAs of the Cas9 orthologs presented in this work

File Name: Supplementary Data 2
Description: Sequences of the oligonucleotides used for cloning sgRNA spacers and sequences of their relative target sites

File Name: Supplementary Data 3
Description: Sequences of all the Cas9 orthologs shown in this work

File Name: Supplementary Data 4
Description: Sequences of the primers used for NGS analysis (PAM identification assay).

File Name: Supplementary Data 5
Description: Parameters employed for the estimation of base editors editing window.

File Name: Supplementary Data 6
Description: Oligonucleotides used to amplify DNA templates to perform TIDE analysis, in vitro cleavage and qPCR.

File Name: Supplementary Data 7
Description: Plasmid sequences for AAV production.
