# Supplementary material for: CoCas9 is a compact nuclease from the human microbiome for efficient and precise genome editing
Source: Nat Commun. 2024 Apr 24;15:3478. doi: 10.1038/s41467-024-47800-9 (PMC11043407; doi:10.1038/s41467-024-47800-9)
Supplement: Supplementary file 11 — Reporting Summary [file 41467_2024_47800_MOESM11_ESM.pdf]

nature portfolio

Corresponding author(s): Anna Cereseto, Nicola Segata

Last updated by author(s): Mar 19, 2024

Reporting Summary

Nature Portfolio wishes to improve the reproducibility of the work that we publish. This form provides structure for consistency and transparency in reporting. For further information on Nature Portfolio policies, see our Editorial Policies and the Editorial Policy Checklist.

Statistics

For all statistical analyses, confirm that the following items are present in the figure legend, table legend, main text, or Methods section.

n/a	Confirmed	
☐	☒	The exact sample size (*n*) for each experimental group/condition, given as a discrete number and unit of measurement
☐	☒	A statement on whether measurements were taken from distinct samples or whether the same sample was measured repeatedly
☐	☒	The statistical test(s) used AND whether they are one- or two-sided<br>*Only common tests should be described solely by name; describe more complex techniques in the Methods section.*
☒	☐	A description of all covariates tested
☐	☒	A description of any assumptions or corrections, such as tests of normality and adjustment for multiple comparisons
☐	☒	A full description of the statistical parameters including central tendency (e.g. means) or other basic estimates (e.g. regression coefficient) AND variation (e.g. standard deviation) or associated estimates of uncertainty (e.g. confidence intervals)
☐	☒	For null hypothesis testing, the test statistic (e.g. *F*, *t*, *r*) with confidence intervals, effect sizes, degrees of freedom and *P* value noted<br>*Give P values as exact values whenever suitable.*
☒	☐	For Bayesian analysis, information on the choice of priors and Markov chain Monte Carlo settings
☒	☐	For hierarchical and complex designs, identification of the appropriate level for tests and full reporting of outcomes
☒	☐	Estimates of effect sizes (e.g. Cohen's *d*, Pearson's *r*), indicating how they were calculated

Our web collection on statistics for biologists contains articles on many of the points above.

Software and code

Policy information about availability of computer code

Data collection	Next-generation sequencing data was collected with Illumina MiSeq.
Data analysis	Analyses were preformed using custom code in Python (3.7), which is publicly available at: https://github.com/Matteo-Ciciani/CoCas9-data-analysis. Additional software used: MinCED version 0.4.2, CRISPRCasTyper v1.2.1, BLAST version 2.2.31, RNIE 0.01, RNAsubopt version 2.4.14, mafft version 7.490, MMseqs2 version 13.45111, IQ-TREE version 2.0.3, GraPhlAn version 1.1.3, Logomaker version 0.8, TIDE version 3.3.0 (http://shinyapps.datacurators.nl/tide/), EditR version 1.0.0 (http://baseeditr.com), guideseq v1.0.2, CRISPResso2 v2.2.10, GraphPad Prism version 9.4.1.

For manuscripts utilizing custom algorithms or software that are central to the research but not yet described in published literature, software must be made available to editors and reviewers. We strongly encourage code deposition in a community repository (e.g. GitHub). See the Nature Portfolio guidelines for submitting code & software for further information.

Data

Policy information about availability of data

All manuscripts must include a data availability statement. This statement should provide the following information, where applicable:

- Accession codes, unique identifiers, or web links for publicly available datasets
- A description of any restrictions on data availability
- For clinical datasets or third party data, please ensure that the statement adheres to our policy

Sequencing data for PAM determination assays, GUIDE-Seq experiments and deep sequencing of mouse retinas are publicly available at NCBI Sequence Read Archive (PRJNA1088104) [https://www.ncbi.nlm.nih.gov/sra/PRJNA1088104]. Source data are provided with this paper.

Research involving human participants, their data, or biological material

Policy information about studies with human participants or human data. See also policy information about sex, gender (identity/presentation), and sexual orientation and race, ethnicity and racism.

Reporting on sex and gender	Sex and geneder of participants were not collected.
Reporting on race, ethnicity, or other socially relevant groupings	Race, ethnicity, or other socially relevant groupings were not collected.
Population characteristics	Population characteristics were not collected.
Recruitment	Not applicable.
Ethics oversight	Written informed consent was obtained from all subjects. All experiments were performed in accordance with the Declaration of Helsinki. The study was approved by the regional investigational review board (reference DC2022-5364, CPP Île-de-France II "Hôpital Necker Enfants malades", Paris, France).

Note that full information on the approval of the study protocol must also be provided in the manuscript.

Field-specific reporting

Please select the one below that is the best fit for your research. If you are not sure, read the appropriate sections before making your selection.

☒ Life sciences ☐ Behavioural & social sciences ☐ Ecological, evolutionary & environmental sciences

For a reference copy of the document with all sections, see nature.com/documents/nr-reporting-summary-flat.pdf

Life sciences study design

All studies must disclose on these points even when the disclosure is negative.

Sample size	A size of three or more was used for all experiments, as a minimum of three independent samples are required to perform statistical tests. This choice was based on previous literature in the genome editing field (e.g. doi.org/10.1038/s41551-022-00911-4; doi.org/10.1038/s41467-023-41829-y; doi.org/10.1038/ncomms14500). No other methods were used to determine sample size.
Data exclusions	No data were excluded.
Replication	All experiments were independently replicated a minimum of three times.
Randomization	Edited samples were compared to untreated (mock) controls. The same cell population was used for treatment or control so there was no bias in the assignment of treatments. The downstream analyses were objective measurements not subject to bias. For in vivo studies involving hRHO-P23H-RFP+/- mice, right and left eyes were randomly assigned to each treatment group.
Blinding	Blinding was not performed for experiments on human cell lines and primary cells. The analyses were objective measures not subject to bias. For all animal experiments, the investigators extracting total DNA and assessing genome editing did not have access to group allocation until after results were obtained.

Reporting for specific materials, systems and methods

We require information from authors about some types of materials, experimental systems and methods used in many studies. Here, indicate whether each material, system or method listed is relevant to your study. If you are not sure if a list item applies to your research, read the appropriate section before selecting a response.

Materials & experimental systems

n/a	Involved in the study
☐	☒ Antibodies
☐	☒ Eukaryotic cell lines
☒	☐ Palaeontology and archaeology
☐	☒ Animals and other organisms
☒	☐ Clinical data
☒	☐ Dual use research of concern
☒	☐ Plants

Methods

n/a	Involved in the study
☒	☐ ChIP-seq
☐	☒ Flow cytometry
☒	☐ MRI-based neuroimaging

Antibodies

Antibodies used	Primary antibodies: mouse anti-V5 antibodies (1:1000 dilution, Thermo Fisher Scientific, #46-0705, clone SV5-Pk1); mouse anti-α-Tubulin antibodies (1:6000 dilution, Sigma Aldrich, #T6074, clone B-5-1-2); mouse anti-GAPDH (1:4000 dilution, Santa Cruz Biotechnology, #sc-32233, clone 6C5); rabbit anti-H3 antibodies (1:10000 dilution, Abcam, #ab1791).<br>Secondary antibodies: goat anti-Mouse (1:15000, dilution, KPL, #0741809) or goat anti-Rabbit (1:10000 dilution, Santa Cruz Biotechnology, #sc-2004).
Validation	All antibodies listed above were purchased commercially and validated by suppliers for westren blot (Thermo Fisher, Sigma Aldrich, Santa Cruz Biotech and Abcam).

Eukaryotic cell lines

Policy information about cell lines and Sex and Gender in Research

Cell line source(s)	HEK293T cells were obtained from ATCC (#CRL-11268), U2OS.EGFP cells are a kind gift of Claudio Mussolino (University of Freiburg), and AAVpro-293T were obtained from Takara (#632273). HEK293-RHO-EGFP cells were obtained by stable transfection of HEK293 (ATCC #CRL-1573) cells with a pcDNA5/TO-RHO-EGFP reporter plasmid. Primary cells: HSF (GM05659) were obtained from the Coriell Institute, HBE (BE121) from the Italian Cystic Fibrosis Research Foundation, non-mobilized human adult HSPCs were obtained from patients with sickle cell disease and mobilized human adult HSPCs from healthy donors patients at the "Hôpital Necker Enfants malades" (Paris)
Authentication	Cell lines were authentication by STR profiling by the providers.
Mycoplasma contamination	Cell lines are routinely verified for mycoplasma by internal department service. All cell lines resulted negative for mycoplasma contamination.
Commonly misidentified lines<br>(See ICLAC register)	No commonly misidentified cell lines were used in this study.

Animals and other research organisms

Policy information about studies involving animals; ARRIVE guidelines recommended for reporting animal research, and Sex and Gender in Research

Laboratory animals	The hRHO-P23H-RFP gene knock in mice are reported by Robichaux et al (Dis Model Mech 2022) were obtained by gene targeting in the Hprt−/− ES cell line AB2.2 123, which was derived from mouse strain 129SvEv . The hRHO-P23H-RFP+/− mice were obtained by crossing hRHO-P23H-RFP−/− with C57BL/6J mice. 5 weeks-old mice were treated with AAV8 via subretinal injection and were sacrificed 4 weeks post-injection to evluate editing efficiency.
Wild animals	No wild animals were used in this study.
Reporting on sex	Female and male mice were considered equivalent and randomly assigned to treatment groups.
Field-collected samples	No field-collected samples were used in this study.
Ethics oversight	Association for Research in Vision and Ophthalmology Statement for the Use of Animals in Ophthalmic and Vision and with the Italian Ministry of Health regulation for animal procedures (Ministry of Health authorization number: 252/2022-PR)

Note that full information on the approval of the study protocol must also be provided in the manuscript.

Plants

Seed stocks: No plants were used in this study.

Novel plant genotypes: No plants were used in this study.

Authentication: No plants were used in this study.

Flow Cytometry

Plots

Confirm that:

- [ ] The axis labels state the marker and fluorochrome used (e.g. CD4-FITC).
- [ ] The axis scales are clearly visible. Include numbers along axes only for bottom left plot of group (a 'group' is an analysis of identical markers).
- [ ] All plots are contour plots with outliers or pseudocolor plots.
- [ ] A numerical value for number of cells or percentage (with statistics) is provided.

Methodology

Sample preparation: Cells were collected by trypsinization, washed in PBS, fixed in formaldehid and analyzed by FACS.

Instrument: FACSCanto (Becton Dickinson)

Software: Software associated with the FACScanto cytometer

Cell population abundance: 10000

Gating strategy: The gating was defined by using EGFP positive controls (U2OS-EGFP not treated with Cas nucleases).

- [ ] Tick this box to confirm that a figure exemplifying the gating strategy is provided in the Supplementary Information.
